# Expression of *Bacteroides fragilis* hemolysins in vivo and role of HlyBA in an intra-abdominal infection model

**DOI:** 10.1002/mbo3.76

**Published:** 2013-02-26

**Authors:** Leandro A Lobo, Audrey L Jenkins, C Jeffrey Smith, Edson R Rocha

**Affiliations:** 1 Department of Microbiology and Immunology, East Carolina University Brody School of MedicineGreenville, North Carolina; 2 Department of Medical Microbiology, Institute of Microbiology, Federal University of Rio de JaneiroRio de Janeiro, Brazil; 3 Department of Comparative Medicine, East Carolina University Brody School of MedicineGreenville, North Carolina

**Keywords:** Anaerobic infection, *Bacteroides fragilis*, experimental intra-abdominal infection, hemolysins, in vivo gene expression

## Abstract

*Bacteroides fragilis* is the most frequent opportunistic pathogen isolated from anaerobic infections. However, there is a paucity of information regarding the genetic and molecular aspects of gene expression of its virulence factors during extra-intestinal infections. A potential virulence factor that has received little attention is the ability of *B. fragilis* to produce hemolysins. In this study, an implanted perforated table tennis “ping-pong” ball was used as an intra-abdominal artificial abscess model in the rat. This procedure provided sufficient infected exudate for gene expression studies in vivo. Real-time reverse transcription polymerase chain reaction (RT-PCR) was used to quantify the relative expression of *hlyA*, *hlyB*, *hlyC*, *hlyD*, *hlyE*, *hlyF*, *hlyG*, and *hlyIII* mRNAs. The *hlyA* mRNA was induced approximately sixfold after 4 days postinfection compared with the mRNA levels in the inoculum culture prior to infection. The *hlyB* mRNA increased approximately sixfold after 4 days and 12-fold after 8 days postinfection. Expression of *hlyC* mRNA increased sixfold after 1 day, 45-fold after 4 days, and 16-fold after 8 days postinfection, respectively. The *hlyD* and *hlyE* mRNAs were induced approximately 40-fold and 30-fold, respectively, after 4-days postinfection. The *hlyF* expression increased approximately threefold after 4-days postinfection. *hlyG* was induced approximately fivefold after 4 and 8 days postinfection. The *hlyIII* mRNA levels had a steady increase of approximately four-, eight-, and 12-fold following 1, 4, and 8 days postinfection, respectively. These findings suggest that *B. fragilis* hemolysins are induced and differentially regulated in vivo. Both parent and *hlyBA* mutant strains reached levels of approximately 3–8 × 10^9^ cfu/mL after 1 day postinfection. However, the *hlyBA* mutant strain lost 2 logs in viable cell counts compared with the parent strain after 8 days postinfection. This is the first study showing HlyBA is a virulence factor which plays a role in *B. fragilis* survival in an intra-abdominal abscess model.

## Introduction

*Bacteroides* spp. are among the predominant members of the human colonic microflora which typically reach 10^11^ colony-forming units (cfu) per gram of stool. They account for about 30–40% of total bacteria found in the colon where at least 500–1000 different species have been so far reported (Savage 1977; Gibson and Roberfroid 1999; Hooper et al. 2002; Eckburg et al. 2005; Smith et al. 2006; Reading and Kasper 2011). *Bacteroides vulgatus* and *Bacteroides thetaiotaomicron* are in general the most common species isolated from fecal samples. The contribution of this predominant group of bacteria in the large intestine is related to a variety of physiological functions. As an example, *Bacteroides* spp. are directly involved in several beneficial processes, such as complex polysaccharide degradation, protection of the gut epithelia from colonization by pathogenic bacteria, development of the intestinal tract, maturation of the mucosal and systemic immune systems, bile acid turnover metabolism, energy harvesting, proteolytic activity, and transformation of toxic and mutagenic compounds (Bernalier et al. 1999; Gibson and Roberfroid 1999; Hooper et al. 2002; Bäckhed et al. 2004; Eckburg et al. 2005; Smith et al. 2006; Turnbaugh et al. 2006; Neu et al. 2007; Tappenden and Deutsch 2007; Wexler 2007; Neish 2009; Sekirov et al. 2010; Reading and Kasper 2011).

However, this symbiotic relationship is not always beneficial. In general, *Bacteroides* opportunistic infections occur as a consequence of a disruption in the integrity of the intestinal mucosa wall resulting from conditions such as gastrointestinal surgery, perforated or gangrenous appendicitis, perforated ulcer, diverticulitis, trauma, perforated colon cancer, and inflammatory bowel diseases (Johnson et al. 1997; Edmiston et al. 2002). Following initial bacterial peritoneal contamination, the host defenses – lymphatic clearance, phagocytosis, and sequestration by fibrin – rapidly clear the bacteria within minutes via lymphatic system and exposed them to systemic defenses (McClean et al. 1994; van Till et al. 2007; Mazuski and Solomkin 2009). While most of bacteria are cleared by host defenses, *Bacteroides fragilis* emerges as the most prevalent anaerobic organism in human infections (Finegold and George 1989; McClean et al. 1994; Mazuski and Solomkin 2009; Park et al. 2009). Despite being only 1% or less of the *Bacteroides* that colonize the human colon, *B. fragilis* is by far the most frequent anaerobe isolated from anaerobic infections. It accounts for about 50–70% of all anaerobes isolated from human infections such as intra-abdominal abscesses, peritonitis, infections of the female genital tract, deep wounds, brain abscesses, and bacteremia (Brook 1989; Finegold and George 1989; Brook and Frazier 2000; Mazuski and Solomkin 2009; Park et al. 2009). The virulence of *B. fragilis* is highlighted by its high frequency of recovery from blood cultures compared with other species of the genus. The incidence of anaerobes is 1–17% of all positive blood cultures (Brook 2010) and the *B. fragilis* group accounts for 45% to 65% of nosocomial and community acquired anaerobic bacteremia (Salonen et al. 1998; Blairon et al. 2006). *Bacteroides fragilis* alone makes up half to two thirds of the *B. fragilis* group species isolated from patients with anaerobic bacteremia (Nguyen et al. 2000; Blairon et al. 2006; Brook 2010). Although this incidence of anaerobic bacteremia is relatively low compared with all bacteremias, it is associated with a high mortality rate (Wilson and Limaye 2004; Cheng et al. 2009; Park et al. 2009; Yoshino et al. 2012) of 16% to 45% mortality (Nguyen et al. 2000; Cheng et al. 2009; Park et al. 2009; Yoshino et al. 2012). Several factors have to be taken in consideration when assessing the pathogenic potential of an organism. With regard to the cases of fatal bacteremia, *B. thetaiotaomicron* and *Bacteroides* (*Parabacteroides*) *distasonis* have a higher (38–100% and 50%, respectively) mortality rate compared with *B. fragilis* (24–31%) (Brook 1990). Nonetheless, *Bacteroides* spp. bacteremia was independently associated with a nearly fivefold increase in relative risk of death (Redondo et al. 1995).

The success of *B. fragilis* pathogenicity is still not completely understood but virulence factors such as capsular polysaccharides, microbial adherence, production of proteases, neuraminidase, an enterotoxin in some strains, lipopolysaccharides, inhibition of phagocytosis, iron acquisition, and resistance to oxidative stress play an important role (Smith et al. 2006; Wexler 2007). The most studied *B. fragilis* virulence factor associated with pathogenicity to date is the production of capsular polysaccharide complexes (CPC) and the abscess formation (Gibson et al. 1998; Krinos et al. 2001). However, strains lacking CPC are still pathogenic in animal models suggesting that unknown virulence factors still remain to be determined (Jotwani and Gupta 1991; Jotwani et al. 1992). There are other factors that, although considered of importance in infections by aerobes and facultative bacteria, have been given little attention as far as their role in the pathogenesis of *B. fragilis* group is concerned. One such mechanism is the ability of *B. fragilis* to produce hemolysins/cytolysins. Microbial hemolysins/cytolysins are considered virulence factors that provide an advantage to invading microorganisms due to their cytotoxic activity of targeting and damaging cell membranes (Schindel et al. 2001; Aldick et al. 2007). One of their main functions is to kill leukocytes and enhance bacterial survival by weakening the host immune response and giving access to nutrients. In this regard, *B. fragilis* 638R contains eight genes homologous to hemolysins but, despite their virulence potential, their roles and contributions to *Bacteroides* pathophysiology in extra-intestinal survival and infection remain unclear (Robertson et al. 2006).

In this context, it is relevant to demonstrate whether *B. fragilis* hemolysins are expressed in vivo and what role they might play in an experimental model of infection. Experimental infections with *B. fragilis* have been carried out by many investigators using mice, rats, rabbits, and guinea pigs as animal models (Onderdonk 2005). These studies have dealt mostly with the ability of *B. fragilis* to form abscesses, immune system responses, and evaluation of antibiotic therapy, but little is known about *B. fragilis* gene expression and regulation during extra-intestinal infection in animal models. Thus, we have adapted the use of the large rat tissue cage model of infection developed by Bamberger et al. (2002) as model to study *B. fragilis* gene expression in vivo. We have quantified *B. fragilis* hemolysin expression by real-time reverse transcription polymerase chain reaction (RT-PCR) to establish reliable and reproducible methods for the detection of *B. fragilis* mRNA within the intraperitoneal cavity. We have focused primarily on the role of the dual-hemolysin components HlyBA for two reasons: first, it seems to be the major hemolysin conferring hemolytic activity in *B. fragilis* 638R in vitro assays (Robertson et al. 2006). Second, the regulation of *hlyBA* mRNA expression in a Fur-dependent manner is suggestive that this dual hemolysin might be required in conditions of iron restriction such as it occurs in host tissues (Robertson et al. 2006). We hope that these studies may contribute to our understanding of the pathogenic mechanisms that make this opportunistic organism to emerge as the number one anaerobic pathogen in human diseases.

## Materials and Methods

### Strains and growth conditions

The strains used in this study are listed in Table 1. *Bacteroides fragilis* strains were routinely grown on BHIS (brain heart infusion supplemented with l-cysteine, hemin, and NaHCO_3_). Rifamycin (20 μg/mL), gentamicin (100 μg/mL), tetracycline (5 μg/mL), and erythromycin (10 μg/mL), were added to the media when required. For some experiments, strains were grown in chopped meat medium supplemented with 0.5% glucose, l-cysteine, hemin, and NaHCO_3_. *Escherichia coli* strains were inoculated in Luria Bertani media supplemented with 100 μg/mL ampicillin, 30 μg/mL kanamycin, or 25 μg/mL chloramphenicol.

**Table 1:** Strains and plasmids used in this study

Strains	Phenotype	References
*Bacteroides fragilis*		
638R	Clinical isolate, Rif	Privitera et al. 1979;
BER-41	638R Δ*hlyBA::tetQ*, Rif, Tet	Robertson et al. 2006;
BER-45	638R Δ*hlyIII::cfxA*, Rif, Cfx	Robertson et al. 2006;
BER-47	638R Δ*hlyBA::tetQ*, pER-80, Rif, Tet, Erm	This study
*Escherichia coli*		
DH10B	Cloning host strain	Invitrogen
Rosetta(DE3)pLys	Host strain for expression of pET systems encoding recombinant protein	Novagen
Plasmids		
pET26b(+)	Expression vector of target genes under control of the T7 promoter and C-terminal fusion of His-tag to the target protein	Novagen
pFD340	A *Bacteroides*–*E. coli* expression shuttle vector, (Amp), Erm	Smith et al. 1992;
pER-18	2035-bp DNA fragment amplified from 638R chromosome containing *hlyBA* ORFs was cloned into the BamHI/SstI sites of pUC19	Robertson et al. 2006
pER-80	A 2035 bp BamHI/SstI promoterless *hlyBA* DNA fragment from pER-18 was cloned into the BamHI/SstI sites of the expression vector pFD340	This study
pER-91	An 816 bp *hlyB* DNA fragment amplified from *B. fragilis* 638R chromosome was cloned into the NdeI/XhoI sites of pET26b(+)	This study
pER-92	A 1011 bp *hlyA* DNA fragment amplified from *B. fragilis* 638R chromosome was cloned into the NdeI/XhoI sites of pET26b(+)	This study

Erm, erythromycin resistance; Cfx, cefoxitin resistance; Rif, rifampicin resistance; Tet, tetracycline resistance; Amp, ampicillin resistance. Parenthesis indicates antibiotic resistance expression in *E. coli*.

### Animal model of infection

The large rat tissue cage model, which consists of intraperitoneal implantation of the perforated table tennis “ping-pong” ball, to establish an intraperitoneal abscess infection was modified from Bamberger et al. (2002). Experiments were performed with 33 male Sprague Dawley rats (weight, >400 g) obtained from Charles River Laboratories International, Inc. (Wilmington, MA). The rats were anesthetized and a single ethylene oxide sterilized table tennis ball with approximately 250–300 1.5-mm diameter holes was surgically implanted in the peritoneal cavity and followed by 4 to 5 weeks for encapsulation. During this period, the rats were maintained in standard husbandry procedures. In this model, the ball became encased in connective tissue, developed blood supply, and became filled with sterile fluids with the appearance of serum similar to the same characteristics described by Bamberger et al. (2002). Following 4–5 weeks, *B. fragilis* strains grown overnight in chopped meat media were diluted in BHIS broth to ≍10^8^ cfu/mL and this suspension was used for both in vitro RNA extraction and inoculate (4 mL) into the encapsulated tissue cage. Samples from the peritoneal cavity cage were aspirated at indicated time points for cfu counts and RNA extraction described below. All procedures involving animals followed the guidelines given by the National Institutes of Health Guide for the Care and Use of Laboratory Animals and approved by the Institutional Review Board and Animal Care and Use Committee of East Carolina University.

### Bacterial viable counts in the abscess content

After the inoculation, 0.3–0.4 mL fluid was drawn from the tissue cage balls at intervals of 1, 4, 8, 15, and 22 days postinfection. A 100 μL aliquots of the aspirated fluids were serially diluted in BHIS broth and dilutions were plated on BHIS incubated in anaerobic chamber incubator at 37°C. After 4–5 days of incubation, cfu/mL was determined. Plates were also incubated aerobically as control for possible abscess contamination.

### Total RNA extraction and real-time RT-PCR

The exudates were aspirated (2–3 mL) from the implanted tissue cage balls at the time points indicated above and immediately mixed with RNAlater (Ambion Inc., Austin, TX) at 1:2 ratio. Then a differential lysis using 0.1% sodium deoxycholate was used to lysis host cells. Sodium deoxycholate at 0.2% was used in some extractions. Bacterial pellet and debris were washed once with phosphate buffer saline (PBS; 50 mmol/L phosphate buffer, pH 7.4, 150 mmol/L NaCl):RNAlater solution at 1:2 ratio containing 0.1% sodium deoxycholate. Total RNA was extracted from the pellet using the hot phenol method described previously (Rocha and Smith 1997) and stored in 100% formamide at −70°C. Total RNA was cleaned using the RNeasy Mini kit (Qiagen, Valencia, CA) according to manufacturer instructions. RNA was DNAse treated using the Ambion DNA-free protocol (Ambion, Inc.). First strand cDNA synthesis was carried out from 1 μL total RNA at 1 μg/μL with random hexamer primers and Superscript III RT kit (Invitrogen Inc., Carlsbad, CA) according to manufacturer's instructions. Real-time PCR quantification of each hemolysin mRNA was performed with 1 μL cDNA sample diluted 1:10 and forward and reverse primers described in Table S1. Real-time PCR efficiencies were performed for each primer set. Total RNA obtained from the bacterial inoculum suspension was used as in vitro control for gene expression. Data were analyzed using the Relative Expression Software Tool (REST) 2008 V2.0.7 for group-wise comparison and statistical analysis of relative expression level results in real-time PCR (Pfaffl et al. 2002). In vivo fold induction of each hemolysin was correlated to their respective fold induction of expression in the inoculum culture in vitro. The 16S rRNA was used as reference to normalize gene expression to a housekeeping gene.

### Construction and purification of HlyA-His_6_-Tag and HlyB-His_6_-Tag recombinant proteins

A 1011 nt DNA fragment encoding the entire *hlyA* open reading frame (ORF) was amplified by PCR using the primer HlyA-NdeI-FOR (CTAACAAAG**C**ATATGGAAG) and HlyA-XhoI-REV (ATCGCA**CTCGAG**ACGGGAACAGTCTTCC). The amplified fragment was cloned in-frame into the NdeI/XhoI sites of pET26b(+) to construct the HlyA-His_6_-Tag C-terminus (rHlyA) recombinant protein expression vector, pER-92. The *hlyB* ORF gene was PCR amplified using the primer HlyB-NdeI-FOR (CTAAAA**C**ATATGGCTGACG) and HlyB-XhoI-REV (AATCTG**CTCGAG**TTTATACACG). The 816 nt DNA fragment was cloned into the NdeI/XhoI sites of pET26b(+) to construct the rHlyB-His_6_-Tag C-terminus (rHlyB) recombinant protein expression vector, pER-91. Bold letters in primer sequences indicate nucleotide modifications to incorporate the necessary restriction sites underlined. Both rHlyA and rHlyB were overexpressed in *E. coli* Rosetta(DE3)pLys and purified by Ni-NTA agarose (Qiagen, Inc., Valencia, CA) affinity chromatography according to the manufacturer's instructions. Eluted fractions were collected and analyzed by SDS-PAGE (sodium dodecyl sulfate polyacrylamide gel electrophoresis). The fractions containing bands of 32 kDa rHlyB or 38 kDa rHlyA were pooled and extensively dialyzed against PBS.

### Genetic complementation of *hlyBA* mutant

A 2035 nt DNA fragment containing the promoterless *hlyBA* gene from *B. fragilis* 638R in pER-18 (Robertson et al. 2006) was digested with BamHI and SstI and cloned into the *Bacteroides*–*E. coli* expression shuttle vector pFD340 (Smith et al. 1992). The new construct, pER-80, was conjugated into BER-41 by triparental mating according to standard protocols (Rocha and Smith 1999). Transconjugants were selected on BHIS plates containing 20 μg/mL rifamycin, 100 μg/mL gentamycin, and 10 μg/mL erythromycin.

### Enzyme-linked immunosorbent assays

High-binding affinity 96-well enzyme-linked immunosorbent assay (ELISA) plates (Corning laboratories) were coated with purified rHlyA A or rHlyB at 4 μg/mL in 50 mmol/L carbonate buffer, pH 9.6. Wells were then washed two times with PBS to remove unbound proteins. Then, wells were blocked with 1% BSA in PBS containing 0.05% Tween 20 (PBS-T). Twofold serial dilutions up to 1:128 of cell-free supernatant of peritoneal exudates collected from the tissue cage model were added to respective wells and incubated at 37°C for 1 h. Plates were washed again in PBS-T. The presence of rat antihemolysin antibodies were detected using goat anti-rat IgG peroxidase conjugates (Southern Biotech, Birmingham, AL). After incubation for 1 h at 37°C and washings, developing solution containing TMB (3,3′,5,5′-tetramethyl-benzidine; Sigma Co., Saint Louis, MO) was added. Reactions were stopped with addition of 2 N HCl and the absorbance of each well was read in a microplate reader Multiskan Ex (Thermo Electron Corporation, Milford, MA) at 450 nm.

## Results

**Figure 1:**
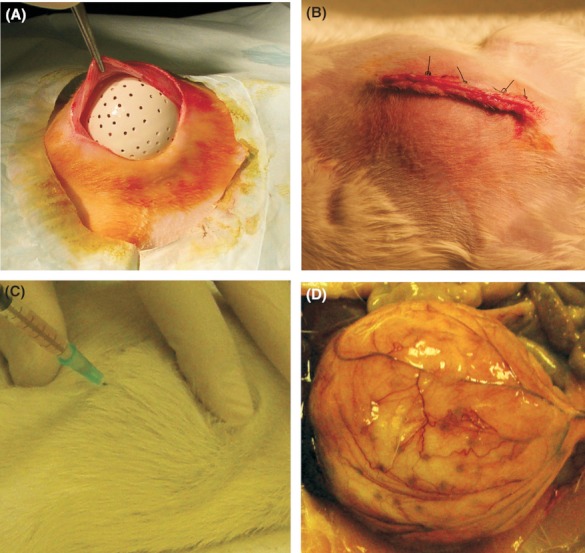
Intraperitoneal tissue cage rat model. A and B) A sterile perforated “tennis ping-pong” ball was implanted into the peritoneal cavity of Sprague Dawley rats (weight, ≥400 g). C) Exudate fluid aspirated from the implanted intraperitoneal perforated tissue cage ball. D) An exposure of the encapsulated perforated tissue cage removed from the peritoneal cavity at the time of necropsy. Panels do not show pictures to scale.

In this study, we show the adaptation of the large rat tissue cage model, which consists of intraperitoneal implantation of the perforated table tennis ball developed by Bamberger et al. (2002), to establish *B. fragilis* intraperitoneal abscess infection. This model of intraperitoneal abscess formation has been successfully used with *Staphylococcus aureus* experimental infections (Bamberger et al. 2002). Consistent with the description of this model, the ball became encased in connective tissue, developed a blood supply, and became filled with sterile fluids with the appearance of serum. In Figure 1, we show representative pictures of the surgery implantation of the tissue cage and the encapsulated cage at the necropsy. The infection process remains confined within the cage. At the time of necropsies, the rats did not shown any signs of systemic or localized infection outside the tissue cage as determined by an independent veterinarian pathologist at the Department of Comparative Medicine. One advantage of this model is that the tissue cage is large enough to allow repeated sampling of an ongoing intra-abdominal abscess infection. This repeated sampling allowed us to obtain *B. fragilis* cells to extract bacterial RNA for gene expression studies from the same inoculated rats in a time course manner. Because very little is known about gene expression of *B. fragilis* in vivo, the expression of hemolysin genes were investigated to determine the usefulness of this model.

### Real-time RT-PCR of hemolysin genes expressed in vivo

**Figure 2:**
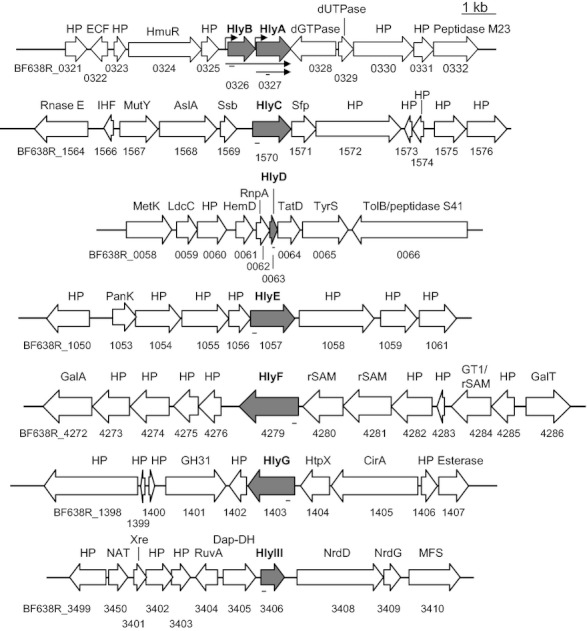
The genetic structure of the *hlyA, hlyB hlyC, hlyD, hlyE, hlyF, hlyG, and hlyIII* loci in the *Bacteroides fragilis* 638R genome. Hemolysins ORF and direction of transcription are depicted by a gray arrow. Open arrows depict ORFs and transcription orientation of the genes flanking each respective hemolysin chromosomal region. The locus tag for each gene and gene product are shown, respectively, below and above each ORF. The transcriptional products for the *hlyBA* operon were adapted from Robertson et al. (2006). The thin dark arrows indicate the direction and length of the *hlyBA* bicistronic mRNA and *hlyA* monocistronic mRNA. The dark bent arrow depicts the putative promoter region derived from Robertson et al. (2006). The bar below each hemolysin ORF represents the coding region and length of the PCR products amplified from cDNAs in the Real-Time RT-PCR experiments described in the Materials and Methods section.

**Figure 3:**
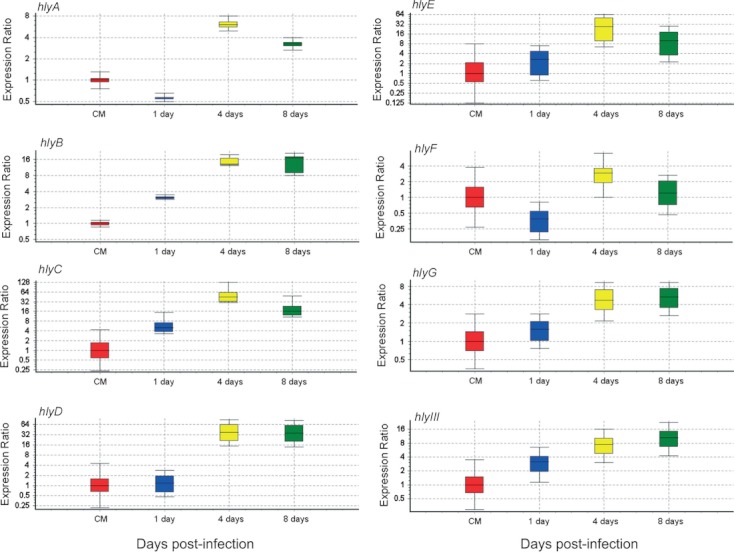
Expression ratio of *B. fragilis* 6389R hemolysins in vivo. Real-time RT-PCR was carried out from total RNA isolated from the intra-abdominal infection tissue cage model implanted into rat peritoneal cavity. Real-time RT-PCR was performed for each hemolysin depicted in each respective panel using specific primers described in the Material and Methods section. *hlyA* expression levels include both *hlyBA* bicistronic mRNA and *hlyA* monocistronic mRNA combined. *hlyB* expression was measured as a component of the *hlyBA* bicistronic mRNA. Data were analyzed using the Relative Expression Software Tool (REST) V. 2.07 for group-wise comparison and statistical analysis of relative expression. In vivo expression of hemolysins was normalized to their respective levels of expression in culture media control (CM). 16S rRNA was used as housekeeping reference RNA. Data are from triplicated real-time RT-PCR reactions in two sets of pooled RNA from two groups of six animals.

The in vivo expression ratio of eight hemolysin genes mRNAs in *B. fragilis* 638R strain was analyzed by real-time RT-PCR and compared with their respective in vitro expression ratio. A diagram of the genetic structural organization for each hemolysin: *hlyA*, *hlyB*, *hlyC*, *hlyD*, *hlyE*, *hlyF*, *hlyG*, and *hlyIII* in the genome of *B. fragilis* 638R is presented in Figure 2. In previous study, we have shown that the *hlyB* and *hlyA* genes are expressed as bicistronic *hlyBA* mRNA but *hlyA* is also expressed as single mRNA (Fig. 2 and Robertson et al. 2006). Therefore, in this study, the real-time RT-PCR for *hlyA* gene expression measured the expression levels of both *hlyBA* mRNA and *hlyA* mRNA combined while *hlyB* transcripts were measured only as *hlyBA* mRNA. In Figure 3, the expression ratio of each hemolysin in the intraperitoneal tissue cage exudate is presented. The *hlyA* transcripts were reduced nearly twofold during the first 24 h postinfection. However, there was an increase of approximately sixfold after 4 days postinfection compared with the mRNA levels in the inoculum culture and remained higher than threefold at 8 day postinfection. The *hlyB* transcripts increased approximately threefold after 1 day, 16-fold after 4 days, and remained elevated at 8 days postinfection. Expression of *hlyC* mRNA increased sixfold after 1 day, 45-fold after 4 days, and 16-fold after 8 days postinfection, respectively. Levels of mRNA for *hlyD* and *hlyE* were not significantly altered after 1 day postinfection but were induced approximately 32-fold after 4-days postinfection. The *hlyF* and *hlyG* mRNAs were not increased significantly after 1 day postinfection and had an approximately three- to fourfold increase after 4 days postinfection, respectively. The *hlyIII* mRNA levels had a steady increase of approximately four-, eight- and 12-fold following 1, 4, and 8 days postinfection, respectively. Overall, it seems that *B. fragilis* hemolysins reached their maximum expression at 4 days postinfection. These findings show that *B. fragilis* hemolysins are induced in vivo and are differentially regulated within the peritoneal cavity at the initial stages of infection.

### Bacterial survival in abscesses

**Figure 4:**
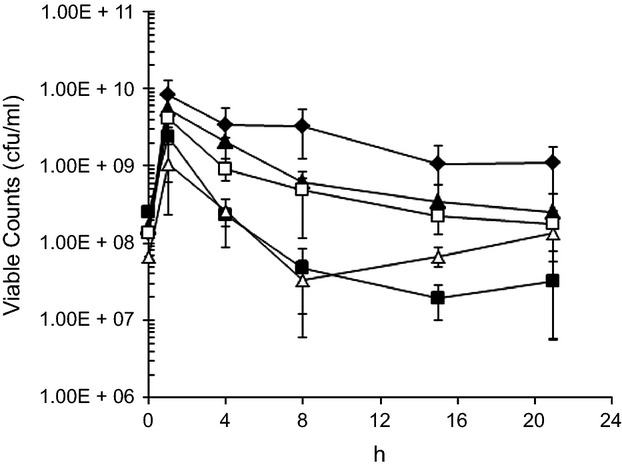
Survival of *B. fragilis* strains inoculated into the encapsulated intraperitoneal implanted tissue cage as described in Figure 1. Fluid exudates were aspirated for viable cell counts (cfu) at time points indicated.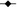: *B. Fragilis* 638R parent strain (*n* = 6).
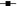
": BER-41 *ΔhlyBA::teQ* deletion mutant (*n* = 6).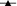: BER-45 *ΔhlyIII::cfxA* deletion mutant (*n* = 3).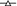: BER-46 *ΔhlyBA::teQ ΔhlyIII::cfxA* double mutant (*n* = 3).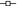: BER-47: *ΔhlyBA::teQ* pER-80 (*n* = 3).

To analyze the role of hemolysin production in the survival of *B. fragilis* in extra-intestinal infection, we looked at the viability of hemolysin mutants: the dual-hemolysin *hlyBA* (BER-41), *hlyIII* (BER-45), and *hlyBA hlyIII* double mutant (BER-46) (Robertson et al. 2006). In the initial 24-h period postinfection, the *B. fragilis* 638R parent strain and hemolysin mutants had a >10-fold increase in cell viable counts compared with the inoculum numbers. However, this was followed by a pronounced decrease in viability of the *hlyBA* and *hlyBA hlyIII* mutants. These two strains lost ≍2 logs in cfu numbers within 4 days postinfection compared with parent strain (Fig. 4). After this period, *hlyBA* mutant survival remained in the range of 1.5 log lower than the parent strain up to 22 days postinfection. *hlyBA hlyIII* mutant also remained lower than the 638R strain. The dual-hemolysin HlyBA alone had the strongest effect on cell survival and additional disruption in the *hlyIII* gene did not have a synergistic effect in *B. fragilis* survival in vivo. Because the hemolytic activity of HlyBA was only determined in heterologous host strain (Robertson et al. 2006), there was no definitive evidence that the HlyBA proteins were the sole responsible for erythrocytes lyses. Here, we show that purified recombinant rHlyA, rHlyB, and the genetic engineered rHlyB:HlyA fusion protein have hemolytic activity against sheep erythrocytes (Data S1). Thus, we believe that the decrease in cell survival in the BER-41 strain is indeed due to the lack of hemolytic activities of the HlyA and HlyB proteins. The *hlyIII* mutant had an apparent intermediate survival rate between the parent strain and the *hlyBA* mutant. When genetic complementation of the dual-hemolysin component HlyBA was constructed in a multicopy plasmid, it only partially restored the survival rate of *hlyBA* mutant in comparison to the survival rates found for the parent strain. This may be due to the fact that extrachromosomal copies of *hlyBA* under constitutive expression promoter control have an adverse effect on bacterial physiological homeostasis.

### ELISA

**Figure 5:**
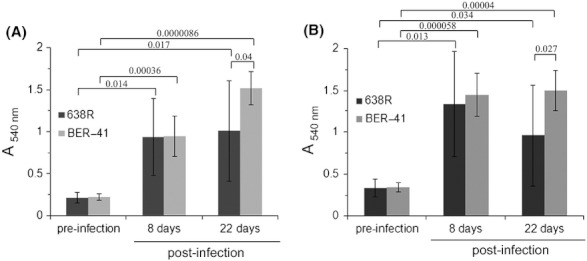
Detection of Rat IgG antibodies reactive against rHlyA and rHlyB by enzyme-linked immunosorbent assay (ELISA**).** IgG levels reactive against rHlyA (panel A) or rHlyB (panel B) in the transudate preinfection (*n* = 3) and exudate fluids (*n* = 3) aspirated from the implanted tissue cage balls at 8 days and 22 days postinfection. Bf 638R: *B. fragilis* 638R parent strain. BER-41 *ΔhlyBA::tetQ* deletion mutant. Microsoft Excel T-TEST software was used to calculate the paired two-tailed Student *t*-test to determine the statistical significance between groups. Group differences were considered statistically significant for *P-*value <0.05. The numbers above horizontal brackets in panels A and B are the probability values associated with the Student *t*-test.

The ELISA was used to assess IgG antibodies level in the uninfected and infected tissue cage fluids. Figure 5 shows the optical density (OD) 450 nm absorbance of the ELISA for anti-HlyA (Fig. 5A) and anti-HlyB (Fig. 5B) reactive antibodies detected at dilutions of 1:128 at 8 and 22 days in the cell-free exudates supernatants compared with the preinfected transudates from intra-abdominal tissue cages. There was a low level of background reactive IgG antibodies against HlyA and HlyB present before bacterial inoculation. This suggests that conventional specific pathogen-free raised rats were sensitized to HlyA and HlyB from *B. fragilis* which is present in the rat normal intestinal microflora. The IgG levels peaked at 8 days for anti-HlyA and anti-HlyB cross-reactive antibodies in tissue cage infected with the parent strain. The levels of anti-HlyA and anti-HlyB antibodies were also elevated in animals inoculated with the *hlyBA* mutant strain. The levels of anti-HlyA and anti-HlyB antibodies were significantly higher in the *hlyBA* mutant strain at 22 days postinfection compared with the levels in the parent strain. It is possible the *hlyBA* mutant strain may be physiologically compensating that lack of *hlyBA* with production of other hemolysins or proteins of the acetyltransferase (pfam1344) and acyltransferase (cd07986) superfamily conserved domains present in HlyA and HlyB (Robertson et al. 2006). In this regard, the alignment of hemolysins deduced amino acid sequences revealed that there are conserved homologous regions which could be potential structural or conformational cross-reactive epitopes (Fig. S1). Thus, we cannot rule out that the elevated levels of IgGs were generated against multiple epitopes of *B. fragilis* antigens cross-reacting with rHlyA or rHlyB. It is also possible that specific antibodies against HlyA and HlyB were present in exudates of rats infected with the parent strain but their levels might have been masked by the robust response of cross-reacting IgGs anti-*B. fragilis*.

## Discussion

In this study, we show the adaptation of the confined large tissue cage rat model to *B. fragilis* experimental intra-abdominal abscess formation. This model was suitable to extract *B. fragilis* RNA for quantification of gene expression in vivo. We used this methodology to determine the expression ratio of *B. fragilis* hemolysins by real-time RT-PCR in a time course postinfection. Our findings show that eight distinct hemolysin genes, *hlyA*, *hlyB*, *hlyC*, *hlyD*, *hlyE*, *hlyF*, *hlyG*, and *hlyIII*, are coordinated and differentially expressed in vivo. This suggests that production of hemolysins may contribute to *B. fragilis* pathogenicity during extra-intestinal infection. Consistent with this, we have also demonstrated that in the absence of the dual-component hemolysin HlyBA, there is an effect on the ability of *B. fragilis* to maintain cell viability at the same levels as the parent strain. In addition, the findings showing that HlyIII had a lesser survival effect than HlyBA indicates that they may have differential role and function in *B. fragilis* in intra-abdominal survival. Despite this modest progress in our understanding, the complete roles of hemolysins in the pathophysiology of this opportunistic pathogen and intestinal colonizer remain to be elucidated.

At this point, we assume that the extensive number of hemolysins in *B. fragilis* offers an advantage to this anaerobe which has a much greater potential to cause infection than any other anaerobic species that colonize the human body. In many Gram-positive and Gram-negative bacteria, production of hemolysins or cytolysins is a powerful virulence factor that results in lysing and killing incoming leukocytes and other host cells (Welch 1991; Rowe and Welch 1994; Menestrina et al. 2003). These features not only promote the survival of the pathogenic microbe by weakening the immune system but also provide access to nutrients (Genco and Dixon 2001; Menestrina et al. 2003). In this regard, it is important to mention that *B. fragilis* is unable to synthesize protoporphyrin macrocycle and has requirement for heme and nonheme iron in vitro and in vivo (Otto et al. 2002; Rocha and Smith 2010). Thus, the expression and regulation of *hlyBA* may promote access to iron and heme in vivo through tissue cells and erythrocytes damage to overcome the iron-limiting conditions imposed by the host's iron-withholding mechanism. This is consistent with our previous report showing that iron-limiting conditions upregulate the expression of the *hlyBA* bicistronic mRNA in a Fur-dependent manner (Robertson et al. 2006). This is also in agreement with iron-limiting dependent regulation mechanism of bacterial hemolysins in many pathogenic bacteria (Braun 1985; Calderwood and Mekalanos 1987; Braun and Focareta 1991; Litwin and Calderwood 1993; Cui et al. 2009; Sineva et al. 2012).

Although very little is known about the mechanisms that may be involved in the regulation of all the *B. fragilis* hemolysins in vivo, one important additional point to be addressed here is the fact that molecular oxygen has a strong inhibitory effect on the expression of both *hlyBA* bicistronic mRNA and *hlyA* monocistronic mRNA (Robertson et al. 2006). These findings may be correlated with the fact that proliferation of *B. fragilis* occurs after the establishment of anaerobic conditions in extra-intestinal infection following reduction of oxygen at the site of infection (Sawyer et al. 1991; Rotstein 1993; Rocha et al. 2007; Sund et al. 2008). In this regard, we assume that the low level of expression of hemolysin, as far as HlyBA is concerned, within the first 24 h postinfection, compared with higher level of expression at 4- and 8-days postinfection, may be indicative of an adaptive period to the transition from aerobic to anaerobic infection mode. These findings agree with recent reports demonstrating that aerotolerance and oxidative stress response are the essential mechanisms for *B. fragilis* proliferation at the initial stages abscess formation in extra-intestinal oxygenated tissues till adequate anaerobic conditions are formed for expression of virulence factors to maintain an infectious process (Rocha et al. 2007; Sund et al. 2008; Reott et al. 2009).

We show the presence of low level of IgG antibodies reactive against *B. fragilis* rHlyA and rHlyB proteins in the transudate aspirated from the encased “ping-pong” ball tissue cage prior to bacterial inoculation as determined by ELISA. It is likely that the adult conventionally raised rats were sensitized to *B. fragilis* that normally colonized the intestinal tract. However, the increase in the levels of IgGs anti-HlyA and anti-HlyB detected in the exudates infected with parent or *hlyBA* deletion mutant strains indicates that these are cross-reactive IgGs antibodies. The rapid and strong increase in IgGs following bacterial inoculation may have triggered secondary systemic immune response to *B. fragilis* multiantigens producing IgGs antibodies that cross-react against HlyA and HlyB. Nonetheless, the upregulation of *hlyBA* mRNA synthesis in vivo confirms that they are produced/secreted in extra-intestinal infections. We assume that the presence of polyclonal antibodies reactive to *B. fragilis* HlyA and HlyB prior to infection may be the result of the possible intestinal mucosal exposure of *B. fragilis* antigens present in the normal intestinal flora in healthy conventional experimental rodents. In experimental infection in adult rabbits, a rapid rise in the levels of IgG antibodies anti-*Bacteroides* antigens has been reported. The inoculation of *B. fragilis* into rabbit sinuses induced rapid and rigorous secondary immune responses initiated by memory T cells that are recruited to the site of pathogen antigen exposure (Jyonouchi et al. 1999). The adult rabbits were likely to be sensitized to *B. fragilis* antigens which is present in the normal rabbit intestinal flora prior to bacteria inoculation (Jyonouchi et al. 1999). The interpretation of our results is further complicated by the fact that membranous structure enveloping tissue implanted devices to create artificial body cavities select for classes of immunoglobulins and serum proteins. In particular, IgG molecules with antibody activity against specific inoculated antigens may be selectively excluded to penetrate in artificial body cavities (Klein et al. 1976). The presence of systemic and localized mucosal immune responses to *B. fragilis* and *B. thetaiotaomicron* antigens colonizing the intestinal tract has being largely reported in both conventional and germ-free monoassociated experimental rodent models (Jyonouchi et al. 1999; Scharek et al. 2000; Peterson et al. 2007) but how the colonic bacterial antigens are presented or exposed to local and systemic immune system through intact mucosal barrier is not completely understood. It is not our objective to discuss at all the complex equilibrium of the inter-relationship between the host mucosal immune system and the intestinal microflora components. For this subject, a wide range of reports are available (Chow et al. 2010; Hooper et al. 2012; Nishio and Honda 2012).

Production of hemolysins/cytolysins by gastrointestinal colonizers and opportunistic pathogens is not only important for systemic infections but also may provide a competitive advantage in the highly competitive intestinal ecological system (Coburn and Gilmore 2003; Cox et al. 2005; Robertson et al. 2006; Rocha and Smith 2010). In this regard, it has been suggested that variations in heme availability in human intestinal tract may affect the growth of *Bacteroides* because of its effect on their short-chain fatty acids fermentation pathway products (Rocha and Smith 2010). Thus, it is possible that *B. fragilis* hemolysins such as HlyA and HlyB may sublethally injure the intestinal mucosal cells to enhance access to this essential heme as nonpathological microbleeding and/or epithelial desquamation are sources of luminal heme (Young et al. 1989). This nonpathological sublethal injury may help the heme-requiring intestinal predominant bacteria such as *Bacteroides* spp. to acquire essential heme. Through this process, it may expose indigenous *Bacteroides* multiantigens to host immune system but this assumption has not yet been tested experimentally.

## Supporting Information

**Table S1.** List of primers used for real-time PCR in this study.

**Data S1.** Construction of recombinant HlyB:HlyA-His6 tag and liquid hemolytic assay.

**Figure S1.** Multiple alignment of the hemolysins deduced amino acid sequence from *Bacteroides fragilis* 638R.
