# Supplementary material for: Expression of Bacteroides fragilis hemolysins in vivo and role of HlyBA in an intra-abdominal infection model
Source: Microbiologyopen. 2013 Feb 26;2(2):326–37. doi: 10.1002/mbo3.76 (PMC3633356; doi:10.1002/mbo3.76)
Supplement: Supplementary file 1 [file mbo30002-0326-SD1.doc]

**Supplemental Material S1**. List of primers used for real-time PCR in this study.

____________________________________________________________________________________

Primer name Nucleotide Sequence

____________________________________________________________________________________

HlyA-forward GGAGGCTATCGGTATATTCTGG

HlyA-reverse CCCGAGTTCAATAGTGGTAGGC

HlyB-forward CGAAAGCTCCGAAGCACTAC

HlyB-reverse ACCTCCAACTTTGCATCCAG

HlyC-forward GTCTTTCATTCGCCTCTTGC

HlyC-reverse ATAGATGACCGGAGCGGAGA

HlyD-forward ACTCCTCCTTCATGTCGTTTCAC

HlyD-reverse CGTCTGACAGCAAGATAAAGTCC

HlyE-forward GGGAATGGAGATTGCTTTTG

HlyE-reverse GTTATTCCCGACCAACATGG

HlyF-forward GGAGAATTGACCCGGTATGA

HlyF-reverse GCCCGTAGTTTGTCACCTTG

HlyG-forward TGATGTGACTCCGCTCTTTG

HlyG-reverse GAGAGCGATGGATTTGGGTA

HlyIII-forward GGCGAAGAGCTGTCCAATAC

HlyIII-reverse CCCCACCAGATAAGCCAGTA

16S-forward GATGCGTTCCATTAGGTTGTTG

16S-reverse CACTGCTGCCTCCCGTAG

**Supplemental Material S2:**

**Construction of fusion HlyB:HlyA-His6-tag recombinant protein:** A 1,860 nt of the *hlyB* and *hlyA* bicistronic operon was amplified by PCR using the primers HlyB-NdeI-FOR and the HlyA-XhoI-REV as described in the Materials and Methods section. The amplified DNA fragment was cloned in-frame into the NdeI/XhoI sites of pET26b(+). The new construct, pER-95 contains the native *hlyB* gene upstream of the *hlyA* fused to the *his_6_-*tag C-terminus of the expression vector. To construct the HlyB protein fused to the HlyA-His6 recombinant protein, a site-direct mutagenesis was performed to replace the *hlyB* stop codon TAA with the glycine codon GGA. This was chosen because this mutation would cause the intergenic 30 nucleotides to be translated in-frame with HlyB and HlyA with 10 amino acids interpeptide fusion region. To accomplish this, pER-95 was amplified by PCR using the complementary primers overlapping the *hlyB* stop codon region, HlyBA-TAA-GGA-FOR (GTGTATAAACTG**GG**ACAGATTAAGG) and HlyB-TAA-GGA-REV (CCTTAATCTGT**CC**CAGTTTATACAC). The bold letters indicate the nucleotides substitution in the stop codon to create the new 273G codon underlined. The circular PCR product was digested with DpnI and transformed into *E. coli* DH10B. The modification in the new construct, pER-96 was confirmed by nucleotide sequence of the mutagenized site and overexpression of the fused protein with predicted MW of approximate 70,000 kDa in Rosetta(DE3)pLys cells. Purification of His_6_-tagged recombinant fused protein was carried out as described in the Materials and Methods section. The recombinant HlyB:HlyA-His6 fused protein (rHlyB:HlyA) forms a single protein homologue to the large hemolysins of *Vibrio anguillarum* and *Wolinella succinogenes* as previously reported (Robertson et al., 2006).

**Liquid hemolytic activity assay:** Cytotoxicity (measured by lysis of erythrocytes) of rHlyA, rHlyB and fused rHlyB:HlyA proteins against sheep red blood cells was determined by the liquid hemolytic assay (Fig. S2). Previous work has shown that cloned native HlyB and HlyA demonstrated synergistic activities in crude extracts of expressing *E. coli* strains (Robertson et al., 2006). Here we show that purified rHlyB and rHlyA proteins at concetrations of 2 nmol per ml had hemolytic activity as 50 % lysis occurred after 4 and 5 hours incubation respectively. We have analyzed whether fused rHlyB:HlyA would retain its functional hemolytic properties as a single large recombinant protein. HlyB is homologue to the N-terminus of *W. succinogenes* large hemolysin and HlyA is homologue to the C-terminal region (Robertson et al., 2006). We chose to replace the *hlyB* stop codon with a glycine codon because the 267G position of *W. succinogenes* hemolysin aligns with the stop codon position in the alignment of amino acid sequences. The fused recombinant rHlyB:HlyA protein at 2 nmol showed hemolytic activity as 50 % lysis occurred about 3 hours of incubation (Fig. S2). We have performed these experiments to show that this reverse phenomenon yielded a functional hemolysin and to suggest that horizontal genetic transfer has occurred. The genetic mutations might have split these two proteins in *Bacteroides* to exert other physiological properties as *hlyBA* deletion mutant has shown changes in colony morphology and growth deficiency (Robertson et al., 2006).


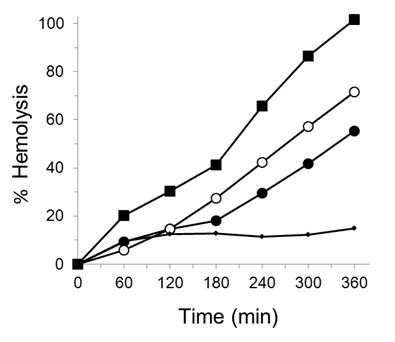


**Fig. S2.** Liquid hemolytic assay. Purified rHlyA, rHlyB and fused rHlyB:HlyA were used to determine hemolytic activity. Sheep erythrocytes were used in the liquid hemolytic assay as target cells. The release of hemoglobin was measured in the supernatant at A_540nm_. Symbols: rHlyA:, rHlyB: , rHlyB:HlyA: , Control blank: . A lysis assay was carried out by mixing 0.5 ml of protein solution at 2 nmol per ml in PBS with o.5 ml erythrocyte suspension (see materials and methods section for details). A mixture of PBS and erythrocyte suspension was used as control blank. Data is the average of two reactions per time point from one experiment.

**Supplemental Material S3:**


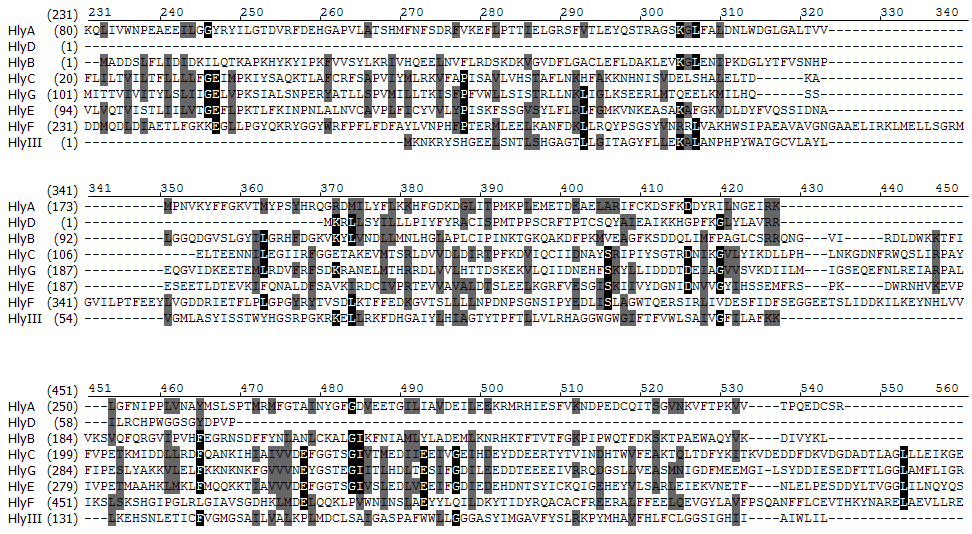


**Fig. S3.** Multiple alignment of the hemolysins deduced amino acid sequence from *Bacteroides fragilis* 638R. HlyA: BF638R_0327, HlyB: BF638R_0326, HlyC: BF638R_1570, HlyD: BF638R_0063, HlyE: BF638R_1057, HlyF: BF638R_4279, HlyG: BF638R_1403 and HlyIII: BF638R_3406. Conserved amino acid residues (>50% identity) are labeled with black boxes. Semi-conserved amino acid substitutions are depicted by grey boxes. Alignment of the peptide sequences was performed using Vector NTI program AlignX V.11.5.2 with peptide score matrix default data file blosum62mt2 for the comparison of amino acid substitution. Only a partial alignment of the most conserved regions is shown.
